# Rise and Recharge: Effects on Activity Outcomes of an e-Health Smartphone Intervention to Reduce Office Workers’ Sitting Time

**DOI:** 10.3390/ijerph17249300

**Published:** 2020-12-12

**Authors:** Abigail S. Morris, Kelly A. Mackintosh, David Dunstan, Neville Owen, Paddy Dempsey, Thomas Pennington, Melitta A. McNarry

**Affiliations:** 1 School of Sport and Exercise Sciences, Swansea University, Swansea SA1 8EN, Wales, UK; a.morris7@lancaster.ac.uk (A.S.M.); k.mackintosh@swansea.ac.uk (K.A.M.); 871889@Swansea.ac.uk (T.P.); 2 Department of Health Research, Faculty of Health and Medicine, Lancaster University, Lancaster LA1 4YW, UK; 3 Baker Heart & Diabetes Institute, Melbourne, VIC 3004, Australia; David.Dunstan@baker.edu.au (D.D.); Neville.Owen@baker.edu.au (N.O.); Paddy.Dempsey@mrc-epid.cam.ac.uk (P.D.); 4 MRC Epidemiology Unit, Institute of Metabolic Science, University of Cambridge, Cambridge Biomedical Campus, Cambridge CB2 0SL, UK; 5 Diabetes Research Centre, University of Leicester, Leicester General Hospital, Leicester LE5 4PW, UK

**Keywords:** feasibility, workplace, intervention, sedentary behaviour, physical activity, sitting, activity breaks

## Abstract

This feasibility study evaluated the effects of an individual-level intervention to target office workers total and prolonged sedentary behaviour during working hours, using an e-health smartphone application. A three-arm (Prompt-30 or 60 min Intervention arm and a No-Prompt Comparison arm), quasi-randomised intervention was conducted over 12 weeks. Behavioural outcomes (worktime sitting, standing, stepping, prolonged sitting, and physical activity) were monitored using accelerometers and anthropometrics measured at baseline, 6 weeks and 12 weeks. Cardiometabolic measures were taken at baseline and 12 weeks. Fifty-six office workers (64% female) completed baseline assessments. The Prompt-60 arm was associated with a reduction in occupational sitting time at 6 (−46.8 min/8 h workday [95% confidence interval = −86.4, −6.6], *p* < 0.05) and 12 weeks (−69.6 min/8 h workday [−111.0, −28.2], *p* < 0.05) relative to the No-Prompt Comparison arm. Sitting was primarily replaced with standing in both arms (*p* > 0.05). Both Intervention arms reduced time in prolonged sitting bouts at 12 weeks (Prompt-30: −27.0 [−99.0, 45.0]; Prompt-60: −25.8 [−98.4, 47.4] min/8 h workday; both *p* > 0.05). There were no changes in steps or cardiometabolic risk. Findings highlight the potential of a smartphone e-health application, suggesting 60 min prompts may present an optimal frequency to reduce total occupational sedentary behaviour.

## 1. Introduction

High levels (6–8 h/day) of sedentary behaviour (SB) are associated with risk factors for chronic diseases and all-cause mortality in adults, with associations remaining even after controlling for time spent in moderate-to-vigorous physical activity [1]. In addition to accumulating recommended levels of at least 150 min of moderate or 75 min of vigorous physical activity weekly [2], current guidelines also recommend that adults minimise the total time spent sitting, including in prolonged periods across all leisure, occupational, transport and household domains [2].

Workplaces in particular have been identified as pivotal settings to target modifiable health behaviours, such as physical activity (PA) and SB, as the majority of daily sedentary time is accrued during working hours [3,4]. Indeed, office and desk-based workers in the UK spend 60–80% of their working hours sedentary, which is not compensated for by increasing PA or decreasing SB during leisure time [5]. Of particular concern is that desk-based workers in the UK spend the majority (52%) of their sedentary time in prolonged bouts (≥30 min), with 25% in prolonged bouts exceeding 55 min [3], a pattern detrimentally associated with musculoskeletal discomfort [6,7], cardiometabolic outcomes [8,9], cerebral blood flow [10] and productivity [11].

Encouragingly, controlled experimental trials suggest that breaking prolonged bouts of sitting with standing or PA is able to ameliorate, or at least attenuate, the acute and chronic deleterious effects. Indeed, frequent interruptions to prolonged sitting bouts with standing appears to be more important for regulating the postprandial glycaemic response compared to solely achieving the minimum PA guidelines [12]. Moreover, multiple interruptions of standing or light PA are more effective at enhancing cardiometabolic health than a single standing or PA break of a longer duration [10,12,13]. There is currently little consensus, however, regarding the optimum break frequency for preventing, or mitigating, the negative health impacts of high sitting time in workplace settings. Additionally, there is a distinct lack of evidence exploring the feasibility of implementing frequent breaks into everyday working practice to address the growing concerns surrounding the amount of time spent in total and prolonged sitting time each day.

While multi-component interventions have been effective for significantly reducing total and prolonged occupational sitting time in desk-based workers over 12 months [14,15], implementation can often incorporate adapting working environments, which can be costly [16]. Alternatively, electronic-health (e-health) interventions delivered via computer software have typically decreased sitting time by 14 and 55 min at 3 and 10 months, respectively [17]. Preliminary evidence suggests that a simple e-health intervention via an activity tracker and mobile application can elicit short-term (3-month) reductions in total daily sitting by an average of 42.4 min/workday [18]. While reductions in total occupational sitting time are smaller compared to multi-component interventions, reductions of more than 30 min per day are potentially clinically meaningful [19] and cost effective [20].

Currently, less is known about the specific role of smartphone e-health technology in promoting a reduction in total and prolonged occupational sitting time compared to computer software-prompting interventions [17]. However, the advancement of wearable activity trackers and e-health smartphone applications offers a potential opportunity to evaluate low-cost and widely accessible alternatives to multi-component workplace interventions [21,22]. Exploring the preliminary intervention effects on behavioural outcomes will provide key insights into the feasibility of implementing different break frequencies within a real-world setting. Thus, the primary aim of this feasibility trial was to evaluate the short-term effects of an individual-level e-health workplace intervention to target office workers total and prolonged sedentary behaviour (using 30 and 60 min prompts) during working hours. The secondary aim was to provide a description and indication of preliminary effects of the secondary outcomes, including anthropometrics and cardiometabolic data, to inform the development of a potentially scalable and cost-effective workplace behaviour change intervention [23,24].

## 2. Materials and Methods

### 2.1. Study Design

Data for this individual-level quasi-randomised feasibility trial were collected over 12 weeks between June and November 2019. The smartphone application used for the intervention was only available on iOS platforms which determined the group allocation according to the make of participant’s mobile device. Specifically, participants with an iOS device were automatically allocated to the Intervention arm and randomly assigned to receive a prompt to break up their sitting time at either 30 or 60 min intervals during working hours (the Prompt-30 and Prompt-60 Intervention arms, respectively); Android users were allocated to a No-Prompt Comparison arm which included a self-monitoring application but provided no prompts to break up sitting during the intervention. The study is reported in line with the Template for Intervention Description and Replication (TIDieR) checklist to enhance transparency and replicability for future trials [25]. Swansea University granted ethics approval (2018-147).

### 2.2. Recruitment of Organisations

Companies with over 50 employees in South Wales were contacted with details of the trial including an overview of the project, timeline, eligibility criteria and data collection measures. Following informal discussions, four companies expressed interest. A subsequent meeting was arranged with the organisational gatekeeper from three of the companies, given they were able to meet the requirements of the study timeline. Gatekeepers consented to support the pragmatic and logistical considerations of the intervention phases and gave permission to contact their employees directly to arrange data collection during working hours. Company One operated in the manufacturing sector across multiple company sites, two of which were approved for the research. Company Two was a University setting, and Company Three was an international software solutions company with an office in South Wales. All companies employed office, and predominately desk-based, employees who represented the convenience sample for the trial.

### 2.3. Recruitment of Office Workers

While there is no specific requirement to conduct a sample size calculation for a feasibility trial, achieving a sample of between 24 and 50 participants has been recommended to estimate a standard deviation for a future sample size calculation for a larger scale trial [26,27,28]. Achieving a sample of 50, however, is deemed suitable to achieve a 0.10 effect size, with a type-I error of α = 0.05, 80% power and a 15% drop out [29]. A sample of this size is suitable for informing future estimates regarding recruitment, retention and attrition to further inform the development of a full randomised controlled trial [30]. Gatekeepers circulated the recruitment email to all staff via an internal mail system during a rolling recruitment period between April and June 2019.

### 2.4. Eligibility and Selection

Participants were screened face to face or via email for the following eligibility criteria: (a) a full-time (or ≥0.6 part-time equivalent) desk-based role; (b) aged 18 years to 64 years; (c) no known cardiovascular/metabolic condition; (d) not pregnant; and (e) owned a smartphone.

### 2.5. Intervention

#### 2.5.1. Theoretical Basis and Intervention Development

The Rise and Recharge intervention was a single-level intervention which aligned with the intrapersonal level within the socioecological model [31,32]. The Intervention arms (Prompt-30 and Prompt-60) involved self-monitoring and prompting behaviour change techniques, whereas the No-Prompt Comparison arm provided an opportunity for participants to self-monitor only [33,34].

#### 2.5.2. Group Allocation and Application Installation

Following baseline assessments and seven-day physical activity monitoring, intervention participants with an iOS device were randomly assigned to the Prompt-30 or Prompt-60 Intervention arms using a random number generator. Installation of the mobile application and on-site data collection made it impossible to blind participants to the intervention they received, or the researchers to the treatment allocation [35]. Participants were emailed a document containing download and user instructions for the application for the purpose of the intervention (Figure S1: Download and user instructions for the Stand Up and Rise and Recharge smartphone applications).

#### 2.5.3. Intervention Application

Once downloaded, the ‘*Stand Up! The Work Break Timer*’ (raisedsquare.com) smartphone application asked participants to select a break frequency. The application had additional features which allowed selection of alternative break frequencies (5–120 min prompts), or to pre-select a minimum duration for a standing break (5–120 min). For the purpose of this trial, however, participants were instructed to select either 30 or 60 min only, and the duration of break was not prescribed and was set to zero as default. Participants were instructed to input their worktimes to ensure that the prompts were only activated during working hours (i.e., Monday–Friday, 08:00–17:00). Work times could be modified throughout the intervention depending on varying work schedules. Throughout the trial, intervention participants received pop-up notifications which stated ‘time to stand up’, accompanied by a sound and/or a vibration alert, on their smartphone at the pre-selected break frequency. To log a break, participants manually clicked the pop-up notification. The application was unable to automatically detect whether a participant was standing, sitting or stepping at the time of the prompt. If a break was missed, or participants were not able to manually enter their break when prompted, this would be classed as a missed break and the next prompt would be automatically scheduled. The application did not remotely store participant information or engagement and adherence to prompts.

Participants in the No-Prompt Comparison arm downloaded the separate Rise and Recharge (Baker Heart and Diabetes Institute) smartphone application, which allowed participants to self-monitor their breaks in sitting time throughout the day. The Rise and Recharge application has an inbuilt function to automatically detect whether a person has taken ≥10 steps in any given 30 min period throughout the day. For participants in the No-Prompt Comparison group, who were using an android device by default, this automatic break detection function was not enabled and instead participants had to manually enter their breaks by clicking on the application and selecting a time (segmented into 30 min slots throughout the day). The Rise and Recharge application provided no prompts to break up sitting time and required participants to manually engage with the application. Participants could not select multiple breaks within a 30 min slot. Once entered, a break would appear on a clock interface within the application and participants could self-monitor accumulation of breaks throughout the day.

### 2.6. Data Collection

Each participant received three emailed calendar invitations directly from the research staff to attend a 1-h assessment at baseline, 6 and 12 weeks. Each 1 h session included anthropometric assessments and participants were fitted with a tri-axial activPAL4 micro monitor (PAL Technologies, Glasgow, UK) and a tri-axial ActiGraph wrist-worn monitor (ActiGraph, Pensacola, FL, USA) to continuously assess SB and PA, respectively, for seven consecutive days. Cardiometabolic health outcomes were measured at baseline and 12 weeks only. Prior to data collection, participants were instructed via email to wear light clothing, avoid the consumption of alcohol and tea and coffee for 12 h, and avoid strenuous exercise for 24 h.

### 2.7. Outcomes

#### Recruitment, Retention and Attrition

**Figure 1:**
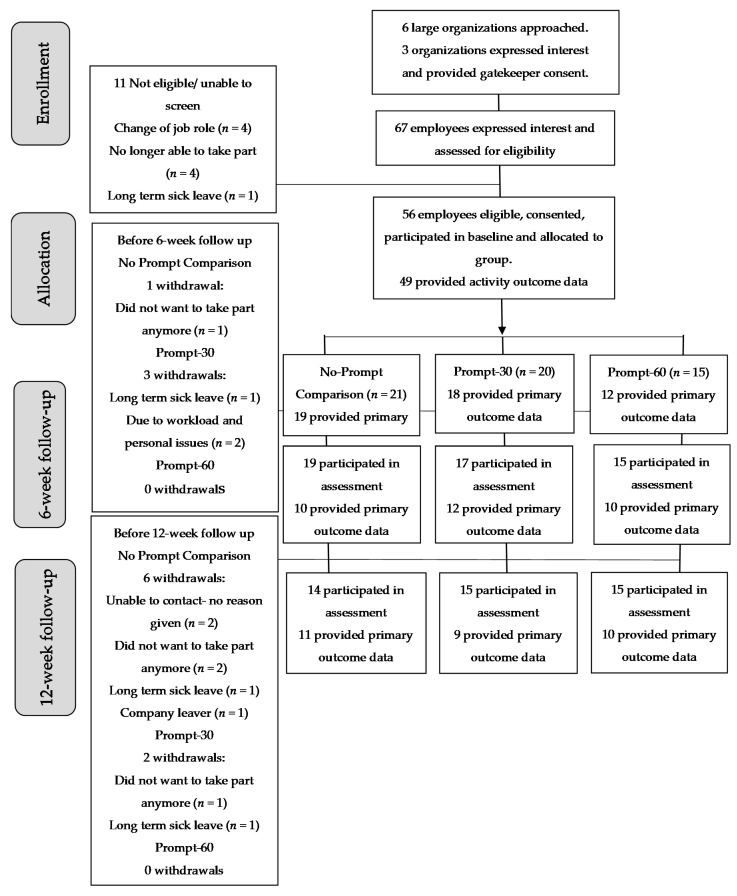
Consort flow diagram of enrolment, allocation, follow-up and analyses.

Participants’ intervention pathway and completion rates for all outcome measures were assessed in line with the guidelines for Consolidated Standards of Reporting Trials [36] (Figure 1).

### 2.8. Behavioural Outcomes

#### 2.8.1. Sitting, Standing and Moving Time

Participant’s work and leisure time spent sitting, standing and stepping, as well as time accrued in sitting bouts ≥ 30 min (prolonged sitting) and total steps taken, were assessed using an activPAL monitor, recorded with a sampling frequency of 20 Hz. The placement of the activPAL was standardised to the anterior midline of the upper right thigh, with monitors inserted into a flexible waterproof sleeve and attached using a hypoallergenic waterproof adhesive strip (Hypafix ^®^, Hamberg, Germany). Participants also wore an ActiGraph GT9X accelerometer sampling at 100 Hz to measure the duration of light, moderate and vigorous PA, and sleep, worn on their non-dominant wrist. To promote wear compliance for both accelerometers and to derive specific work times for further analysis, participants were instructed to report the time they started and finished work (when applicable), went to bed, went to sleep, woke up and got out of bed, in a daily diary [37].

#### 2.8.2. Anthropometry: Stature, Body Mass and Body Composition

Using standard anthropometric techniques [38] and with participants wearing light clothing and no shoes, stature was measured to the nearest 0.1 cm using a portable stadiometer (Seca Ltd., Birmingham, UK) and body mass was measured to the nearest 0.1 kg using a calibrated mechanical flat scale (Seca Clara 803, Seca Ltd., Birmingham, UK). Body mass index was subsequently calculated (kg/m^−2^). Waist and hip measures were measured to the nearest 0.1 cm using an inelastic anthropometric tape (Lufkin W606PM, Apex Tool Group Ltd., Sparks, MD, USA). Waist measurements were standardised to the midline between the iliac crest and the lower rib cage, with participants instructed to breathe normally during assessments. Hip circumference was standardised to the maximum circumference around the gluteals [39]. For all outcomes, if the difference between the first two measures taken exceeded >1%, a third measure was taken and the mean calculated.

#### 2.8.3. Cardiometabolic Markers

In accordance with standardised guidelines [40] and after 15 min of seated rest, an automated sphygmomanometer (Omron, Milton Keynes, UK) was used to measure resting blood pressure on the brachial artery of participant’s bare right arm two times, at one-minute intervals. If the difference between the two measures was ≥5 mmHg, a third measure was taken and the mean calculated. A non-fasted finger prick blood sample was taken using the standard technique to obtain 40 µL samples which were immediately analysed for Hba1c (Hemocue 501, Kuvettgatan, Sweden), total, high-density lipoprotein, and low-density lipoprotein cholesterol (Cardiochek ^®^ Professional Analyser, BHR Pharmaceuticals Ltd., Nuneaton, UK).

#### 2.8.4. Survey Measures and Outcomes

To describe the sample, participants completed an online questionnaire including a non-validated survey adapted from a previous trial [41] to assess sociodemographic (age, gender, ethnicity, marital status, and education), work history (employment history, employment status, job category, hours worked, and main work tasks) and work environment (number of people in their office) characteristics.

### 2.9. Analyses

#### 2.9.1. Behavioural Outcomes

ActivPAL data were downloaded using the manufacturer software (PAL technologies, Glasgow, UK) and processed using ProcessingPAL-V1.2 (Diabetes institute, Leicester, UK), with the first day removed from the analysis to minimise the risk of reactivity to the monitor [37]. The processingPAL software uses a validated algorithm to separate valid waking wear data from sleep, non-wear time or invalid data. A day was considered invalid if there was limited postural variation (i.e., ≥95% of wear time in one activity), limited steps (<500 steps/day) or <10 h valid waking wear time [42]. This algorithm has demonstrated almost perfect (kappa > 0.8 for 88% of participants) agreement with the traditional diary method [42]. Summary data from the algorithm were quality checked using heat maps against participant diaries to check whether the algorithm had successfully been applied to the data [37]. Manual corrections were made if the self-reported waking time was not consistent with the algorithm output [42]. Participants’ workdays and times were manually entered into a .csv template and uploaded into the processingPAL software, which enabled the calculation of worktime PA and SB. Worktimes were considered valid if there was >90% agreement with participants self-reported worktimes. Worktime behavioural outcomes derived from the activPAL data were standardised to an eight-hour working day (average min/8 h workday) and whole day outcomes were standardised to a sixteen-hour day, as reported in previous workplace interventions [15,43]. Participants were included in complete case worktime analyses if they provided ≥1 valid worktime day at baseline, 6 and 12 weeks [14].

Data from the tri-axial GT9X were downloaded using ActiLife version 6.10.4 software (ActiGraph, Pensacola, FL, USA) and saved in raw format as .csv format for data processing [44]. Raw data were processed using the open source R–package GGIR (version 2.0-0) to detect non-wear time, abnormally high accelerations and calculate light-, moderate- and vigorous-intensity PA and sleep according to the acceleration thresholds of Hildebrand et al. [45]. Data were considered valid if there were more than 10 h of valid activity data per day with >90% estimated wear time and recorded >1 valid workday at baseline, 6 and 12 weeks [46].

#### 2.9.2. Statistical Analysis

Descriptive statistics were calculated for sociodemographic, work, PA levels, anthropometric and cardiometabolic outputs (Table 1). In line with the primary aim of this feasibility trial, behavioural data were analysed to determine the preliminary effects of the intervention using linear mixed models in STATA MP (v.13, StataCorp, College Station, TX, USA) to account for the nested nature of the data, with the alpha level set at *p* ≤ 0.05. To control for any imbalances at baseline, baseline values for the variable were controlled for in all analyses [47]. Means and standard deviations for behavioural outcomes are presented by type of day (worktime and whole day), group and time point (Table 2), with adjusted-change coefficients, 95% confidence intervals and standardised effect size (Cohen’s d) between Intervention arms relative to the No-Prompt Comparison (Table 3). Effect sizes were calculated by dividing the difference in group means by the average standard deviation of the pooled data. Data were interpreted as small, medium and large effects for a d value of 0.2, 0.5 and 0.8, respectively [48]. To account for uncertainty in the effect size estimates, 95% confidence intervals were calculated [49,50]. The secondary aim of the feasibility trial was to provide a description and indication of preliminary effects of the secondary outcomes including anthropometrics and cardiometabolic data, and therefore linear mixed model analysis was also conducted following the same procedure outlined above.

## 3. Results

Figure 1 presents the flow of the participants through the intervention between June and November 2019. Overall, 56 participants from three organisations were recruited and assigned to either the No-Prompt Comparison (*n* = 21), Prompt-30 (*n* = 20) or Prompt-60 arms (*n* = 15). The attrition was 7% (No-Prompt Comparison: *n* = 1, 5%; Prompt-30: *n* = 3, 15%; Prompt-60: *n* = 0, 0%) and 14% (No-Prompt Comparison: *n* = 6, 29%; Prompt-30: *n* = 2, 13%; Prompt-60: *n* = 0, 0%) at 6 and 12 weeks, respectively, with 79% (No-Prompt Comparison: *n* = 14, 71%; Prompt-30: *n* = 15, 71%; Prompt-60: *n* = 15, 100%) of total participants retained throughout the trial. There were no withdrawals due to adverse events.

### 3.1. Baseline Characteristics 

At baseline, 6 and 12 weeks, 54 (96%), 44 (76%) and 34 (69%) participants provided at least three valid days of accelerometer data (primary outcome data), respectively (Table S1: Completion rates for outcome measures per time point). Participants were predominantly female (64%), White British, full-time employees, educated to the tertiary level with at least three years tenure (Table 1). On average, at baseline, participants were pre-hypertensive [40], overweight [51], had an elevated waist circumference [52], were sedentary for ≥10 h per day and spent 69% of work hours sitting (of which 64% was spent in prolonged bouts ≥30 min), 24% standing and 7% stepping.

### 3.2. Behavioural Outcomes

Mean worktime behavioural data are presented by the Intervention arm at baseline, 6 and 12 weeks (Table 2). In the complete case analysis (Table 3), the beta coefficients calculated from the linear mixed model analysis indicate a significant difference between groups, in favour of the Prompt-60 Intervention arm, was observed for total worktime sitting at 6 and 12 weeks relative to the No-Prompt Comparison group (*p* = 0.022 and 0.001, respectively). Relative to the No-Prompt Comparison group, there were changes in worktime sitting the Prompt-30 arm at 6 and 12 weeks, although these were not statistically significant. Results indicate that sitting was primarily replaced by worktime standing for Prompt-60 (*p* = 0.032, 0.001) and Prompt-30 at 6 and 12 weeks, respectively. Worktime stepping in Prompt-60 and Prompt-30 were unchanged at 6 or 12 weeks, respectively. Changes to prolonged sitting time, in favour of the Intervention arms, indicate that Prompt-30 and Prompt-60 reduced the amount of time accumulated in prolonged sitting bouts relative to the No-Prompt Comparison arm at 12 weeks. However, these findings were not statistically significant. Results did not differ when controlling for age, gender, company or application use. Overall, preliminary findings indicate small to moderate effects on worktime behavioural outcomes for Prompt-30 and large effects for Prompt-60 across 6 and 12 weeks, with 95% confidence intervals showing large variances across all variables. Descriptive PA results from the ActiGraph GT9X are presented in Supplementary Materials (Table S2: Descriptive PA data from ActiGraph GT9X presented by group and time point).

### 3.3. Secondary Outcomes

Completion rates (calculated as the percentage of participants that provided valid data from those who engaged in data collection per time point) for secondary outcomes ranged from 35 to 100% (Table S1: Completion rates for outcome measures per time point). In line with the secondary aims of this feasibility trial, descriptive statistics for anthropometric results (Table S3: Participants mean anthropometric data collected at baseline, 6 and 12 weeks) and cardiometabolic results (Table S4: Participants mean cardiometabolic data collected at baseline and 12 weeks) are presented.

Preliminary effects on anthropometric outcomes indicate small changes in body mass for Prompt-30 and Prompt-60 at 6 and 12 weeks. Preliminary effects on waist to hip ratio were moderate for Prompt-30 and small for Prompt-60 at 6 and 12 weeks, respectively. Anthropometric results did not indicate statistical significance (Table S5: Adjusted mean change and standardised effect size (Cohen’s d) between the Prompt-30 and Prompt-60 Intervention arms, relative to the No-Prompt Comparison arm for anthropometric outcomes).

Preliminary effects on cardiometabolic outcomes indicate small changes in systolic and diastolic blood pressure, HDL cholesterol, cholesterol ratio and glucose (HBa1C), and moderate changes in total and LDL cholesterol among Prompt-30 participants. These were not statistically significant. Preliminary effects on cardiometabolic outcomes among Prompt-60 indicate small changes in systolic and diastolic blood pressure, total, LDL cholesterol. However, these results were not statistically significant. Moderate changes were found for HDL and cholesterol ratio among Prompt-60 participants, and changes in glucose (HBa1C) indicated statistical significance (*p* = 0.04; Table S6: Adjusted mean change and standardised effect size (Cohen’s d) between the Prompt-30 and Prompt-60 Intervention arms, relative to the No-Prompt Comparison arm for cardiometabolic outcomes). Overall, preliminary findings indicate large variance in effects for cardiometabolic and anthropometric outcomes for Prompt-30 and Prompt-60 participants.

## 4. Discussion

Overall, exploring the preliminary intervention effects on behavioural outcomes aimed to provide key insights into the feasibility of implementing different break frequencies within a real-world setting. In this feasibility trial, we evaluated the short-term effects of an individual-level workplace intervention to target office workers total and prolonged sedentary behaviour during working hours, using an e-health smartphone application. Initial results indicate that the Prompt-60 participants demonstrated greater reductions in their total sitting time at 6 and 12 weeks compared to the Prompt-30 participants. Prolonged sitting time at work was also reduced in both Intervention arms (Prompt-30 and Prompt-60) at 12 weeks. Sitting was largely replaced with standing and there were no changes in the time spent stepping during working hours. These preliminary findings, therefore, highlight the potential utility of an e-health smartphone application for targeting total and prolonged occupational sedentary time, but not PA.

The reduction in sitting time among the Prompt-60 participants was higher than other technology-based intervention studies following 12 weeks (−69.6 min/8 h workday) when standardised to an eight-hour working day [21]. Notably, however, the reduction in sitting time was smaller in the Prompt-30 arm (−36.6 min/8 h workday). This highlights important considerations regarding the influence of the frequency of prompts on total sitting time during working hours, and raises questions regarding the contextual factors which may influence participant adherence to either a 30 or a 60 min prompt. Importantly, the current findings revealed that the pattern in which sitting time was accumulated was also influenced by the smartphone prompts, with both Intervention arms associated with a reduced amount of time spent sitting in prolonged bouts (≥30 min) after 12 weeks, compared to the No-Prompt Comparison arm. This finding is pertinent given office workers high exposure to total and prolonged sitting time at work [3,53] and the detrimental association with prolonged sitting and cardiometabolic [54], psychosocial [55] and musculoskeletal health [7].

The present trial targeted a behavioural intervention at a single level, which encompassed prompts as the primary behaviour change technique [34]. Overall, changes in total and prolonged sitting at work appeared smaller in magnitude compared to other multi-component trials involving a combination of behavioural and environmental components [17,19]. Although importantly, our findings indicate reductions in sitting time greater than 30 min, which is potentially meaningful for health [17,19]. The behavioural observations in the present trial provide some preliminary evidence in support of implementing a simple e-health intervention as a cost-effective, and widely accessible strategy to support office workers to reduce their total and prolonged sitting time [20]. Alternatively, implementing e-health intervention components alongside effective multi-component strategies, such as height-adjustable workstations, may also help to optimise occupational behaviour change by enhancing employees capabilities, opportunities and motivation to sit less and move more [56].

Notably, the intervention message in the present trial did not provide guidance on the duration of break or break modality. Rather, participants were encouraged to transition from a seated posture to either a standing or ambulatory posture when prompted, in line with the current workplace recommendations [57]. Indeed, the lack of intervention message to walk or conduct other light PA or resistance exercises is likely reflected in the lack of effect on overall worktime stepping or PA. However, our preliminary findings appear consistent with other workplace trials to date [17]. While there is currently inconsistent evidence regarding the optimum duration or intensity of breaks for health, there is growing empirical evidence demonstrating that frequent bouts of light PA or simple resistance exercises are more beneficial for improving cardiometabolic health markers than standing breaks alone [10,58,59,60]. The feasibility of implementing frequent breaks consisting of light-to-moderate-intensity activities within real-world office settings, however, is questionable, due to perceived social norms which consider PA as disruptive and unproductive among management and employees [61,62].

Common barriers to reducing sitting time and increasing PA at work have previously been identified as having a high workload and productivity demands, in addition to job demands which are synonymous with seated working postures [63]. Previous studies have also highlighted the importance of harnessing interpersonal factors, such as management support [64] and workplace champions [65], to facilitate a change in attitudes and workplace cultures around typical sitting behaviours. Incorporating interpersonal strategies into future trials therefore appears vital for facilitating opportunities for light PA. In addition, further consideration should be given to the type of intervention messages used in future trials to promote frequent breaks consisting of light or moderate PA.

Overall, the short-term changes to cardiometabolic outcomes appears similar to those observed in a recent meta-analysis of multi-component workplace interventions [66] and indicates small to moderate, changes irrespective of Intervention arm, at 12 weeks. Notably, cardiometabolic changes in both intervention groups occurred following a single-component intervention and may be of importance for future interventions seeking to attenuate cardiometabolic risk factors associated with adverse health outcomes [55]. These results should, however, be interpreted with caution due to the low sample size and lack of statistical power. Nonetheless, our findings provide some preliminary evidence in support of further evaluation of an e-health smartphone intervention to prompt regular breaks from seated work, in line with the current workplace guidelines [59]. Further research should explore whether the observed behavioural changes are beneficial for cardiometabolic health over time when compared to usual practice controls [66].

Given the rapid advancement and accessibility of smart technologies including phone and tablet devices, wearable technologies and computer software, it may be important to determine which mode of delivery is the most effective and favourable for office workers in future trials. The present trial relied on participant engagement with the application in order to log a break or to self-monitor their break activity, while other automatic technologies are available which may minimise this participant burden. To date, comparative technology-based literature is limited and largely involves computer software strategies to prompt behavioural changes [17]. Compared to computer-based interventions alone, smart and wearable devices arguably have the potential to be more effective given their portable appeal, large usage among working-age populations [22] and ability to prompt sitting reductions in a variety of contexts. However, further research is warranted to determine the effectiveness and sustainability of such technologies over time.

### Strengths and Limitations

A strength of the present trial was that it was delivered in real-world workplace settings, enhancing ecological validity. Participant attrition was 7% and 14% at 6 and 12 weeks, respectively, which is lower than experienced within other workplace intervention trials [17]. Furthermore, there were no intervention withdrawals due to adverse events [15]. As highlighted throughout, the feasibility study design was quasi-randomised and was not powered to detect changes in behavioural or cardiometabolic outcomes. Results from this exploratory study should therefore be interpreted with some caution. Our initial findings, however, are encouraging and provide sufficient data to conduct a power calculation to inform the development of a fully powered trial among desk-based office workers, to explore effectiveness and sustainability of behavioural and cardiometabolic changes [27]. Indeed, our findings warrant further investigation using a randomised controlled trial design to establish the effectiveness of the intervention on behavioural outcomes, while controlling for potential risks of bias which could not be mitigated in this quasi-randomised trial design [67].

Due to the e-health application availability on iOS platforms only, this may have introduced a potential bias in the allocation of individuals to either a No-Prompt Comparison or Intervention arm. The authors attempted to minimise allocation bias by randomly allocating participants to the Prompt-30 or Prompt-60 Intervention arms and use statistical analysis techniques which account for any imbalances at baseline. However, due to the quasi-randomised approach, it was not possible to control for heterogeneity among participant groups. Furthermore, participants were often co-located within the same office, or within close working proximity to one another, which may introduce a risk of contamination between Intervention arms. Future trials should therefore attempt to deliver a cluster randomised controlled trial design to mitigate these risks [68]. Furthermore, it is possible that the behaviour of participants in the No-Prompt Comparison arm was influenced by the health check feedback, self-monitoring application, or co-location with intervention participants across the trial. Overall, however, there were no beneficial changes in worktime behavioural outcomes among this Comparison group which would support this. Nevertheless, future trials should include a non-intervention comparison arm and a rigorous study design to determine the effectiveness of the intervention compared to usual practice controls.

## 5. Conclusions

In conclusion, this study highlights the potential utility of a smartphone e-health application for targeting total and prolonged occupational sedentary time. Importantly, the current findings suggest that 60 min prompts may present an optimal frequency to reduce total sedentary behaviour during working hours. Both 30 and 60 min prompts may also be beneficial for reducing prolonged sitting bouts over 12 weeks. Findings will be of value to researchers seeking cost-effective strategies for desk-based employees to reduce sedentary working practices. The challenge is to develop an understanding surrounding the contextual factors influencing office workers’ adherence to sitting breaks at work and to determine whether these behavioural changes are effective and sustainable over time.

## Figures and Tables

**Table 1:** Baseline characteristics of participants (*n* = 56) presented by the Intervention arm.

	All (*n* = 56)	Comparison Attrition (*n* = 20)	Prompt-30 (*n* = 21)	Prompt-60 (*n* = 15)
Female	36.0 (64)	14.0 (70)	13.0 (62)	9.0 (60)
Age (years)	39.8 ± 11.4	43.5 ± 10.5	39.3 ± 13.5	35.9 ± 8.4
White British	47.0 (84)	17.0 (85)	16.0 (76)	14.0 (93)
Married	30.0 (54)	10.0 (50)	15.0 (71)	5.0 (33)
Full-time employee	47.0 (84)	16.0 (80)	16.0 (76)	15.0 (100)
Tenure in current role ≥ 3 years	34.0 (61)	14.0 (70)	12.0 (57)	8.0 (53)
Tertiary education	45.0 (80)	17.0 (85)	13.0 (62)	15.0 (100)
Management job role	16.0 (29)	6.0 (30)	6.0 (29)	4.0 (27)
Clerical, sales, or admin job role	29.0 (52)	11.0 (55)	9.0 (43)	9.0 (60)
Daily hours worked (h/day)	7.6 ± 0.6	7.5 ± 0.5	8.0 ± 1.0	7.5 ± 0.5
Weekly hours worked (h/week)	36.4 ± 4.1	35.5 ± 6.0	37.2 ± 2.8	36.3 ± 5.4
Stature (m)	1.7 ± 0.1	1.7 ± 0.1	1.7 ± 0.1	1.7 ± 0.1
Body mass (kg)	83.0 ± 19.9	87.4 ± 18.3	81.3 ± 18.1	79.8 ± 24.5
Body mass index (kg·m^−2^)	29.0 ± 6.3	31.0 ± 6.8	28.4 ± 5.9	26.9 ± 5.7
Waist circumference (cm)	89.8 ± 16.3	95.4 ± 16.7	88.8 ± 15.4	83.8 ± 15.7
Hip circumference (cm)	107.3 ± 13.3	108.8 ± 16.0	107.4 ± 10.0	105.3 ± 14.3
Systolic blood pressure (mmHg)	135.7 ± 19.2	139.3 ± 16.0	137.7 ± 17.7	132.3 ± 14.5
Diastolic blood pressure (mmHg)	87.3 ± 17.3	91.1 ± 14.8	89.8 ± 7.2	87.5 ± 10.1
Glycated haemoglobin HbA1C (%)	5.6 ± 0.3	5.7 ± 0.4	5.4 ± 0.3	5.6 ± 0.3
Glucose (mmol·L^−1^)	5.16 ± 1.0	5.8 ± 1.0	4.7 ± 0.7	4.9 ± 0.9
Total cholesterol (mmol·L^−1^)	4.26 ± 1.0	4.5 ± 1.2	4.0 ± 0.8	4.2 ± 1.1
High-density lipoprotein (HDL) cholesterol (mmol·L^−1^)	1.16 ± 0.5	1.0 ± 0.3	1.2 ± 0.5	1.2 ± 0.4
Low-density lipoprotein (LDL) cholesterol (mmol·L^−1^)	3.10 ± 1.1	3.4 ± 1.2	2.8 ± 0.8	3.0 ± 1.0
Cholesterol ratio (total cholesterol:HDL)	4.16 ± 2.0	4.8 ± 1.9	3.7 ± 1.6	3.8 ± 2.2
Behavioural worktime (ActivPAL)				
Valid wear (days)	3.3 ± 1.7	3.5 ± 1.6	3.9 ± 1.8	3.5 ± 1.7
Sitting time (min/8 hday)	330.1 ± 89.8	329.4 ± 83.1	320.7 ± 110.5	345.2 ± 67.8
Standing time (min/8 hday)	115.3 ± 87.1	115.4 ± 75.5	125.5 ± 110.4	100.0 ± 67.2
Stepping time (min/8 hday)	34.6 ± 13.9	35.2 ± 14.5	33.79 ± 14.8	34.7 ± 12.5
Prolonged sitting time (≥30 min/8 hday)	211.0 ± 95.3	235.4 ± 96.0	184.3 ± 100.8	212.3 ± 81.5
Steps (number/8 hday)	3138.8 ± 1279.7	3130.6 ± 1178.8	3131.0 ± 1484.3	3163.8 ± 1211.0
Sit–upright transitions (number/8 hworkday)	27.6 ± 12.1	25.3 ± 8.0	30.5 ± 17.4	26.6 ± 6.3
Behavioural whole day (ActivPAL)				
Valid wear (days)	6.0 ± 1.5	5.9 ± 1.6	6.3 ± 1.4	5.8 ± 1.6
Sitting time (min/16 hday)	612.4 ± 101.8	629.9 ± 78.7	589.2 ± 112.6	623.2 ± 96.6
Standing time (min/16 hday)	241.9 ± 81.6	227.1 ± 65.53	266.9 ± 96.4	224.5 ± 73.0
Stepping time (min/16 hday)	105.6 ± 37.2	103.0 ± 33.3	103.9 ± 42.9	112.4 ± 35.0
Prolonged sitting time (≥30 min/16 hday)	325.9 ± 113.4	355.1 ± 103.3	295.8 ± 123.3	329.6 ± 107.4
Steps (steps/day)	4380.6 ± 1743.6	8681.6 ± 3349.1	8413.3 ± 3715.6	9446.0 ± 3493.9

No-Prompt Comparison arm downloaded the Rise and Recharge smartphone application and received no prompts during the intervention. The Prompt-30 and Prompt-60 Intervention arms downloaded the Stand Up smartphone application and installed prompts at either 30 or 60 min intervals, respectively. Data are presented as the *n* (%) or the mean ± SD.

**Table 2:** Mean behavioural data per Intervention arm at baseline, 6 and 12 weeks.

	No-Prompt Comparison			Prompt-30			Prompt-60		
	Baseline	6 Weeks	12 Weeks	Baseline	6 Weeks	12 Weeks	Baseline	6 Weeks	12 Weeks
**Worktime (ActivPAL)** **(min/8 h workday)**									
Sitting Time	329.4 ± 83.1	331.6 ± 97.2	369.5 ± 86.0	320.7 ± 110.5	343.2 ± 81.2	326.0 ± 119.3	345.2 ± 67.8	314.6 ± 112.3	302.4 ± 117.4
Standing Time	115.4 ± 75.5	120.3 ± 96.5	83.1 ± 80.5	125.5 ± 110.4	104.3 ± 74.4	123.1 ± 119.1	100.0 ± 67.2	129.7 ± 115.8	140.9 ± 116.7
Stepping Time	35.2 ± 14.5	28.1 ± 9.6	27.4 ± 10.6	33.8 ± 14.8	32.4 ± 20.6	30.9 ± 14.7	34.8 ± 12.5	35.7 ± 16.9	36.7 ± 17.9
Prolonged Sitting Time (≥30 min)	235.4 ± 96.1	160.1 ± 118.5	218.6 ± 116.2	184.3 ± 100.8	141.5 ± 75.2	139.8 ± 105.2	212.3 ± 81.5	179.7 ± 107.8	153.6 ± 116.9
Steps (number)	3130.6 ± 1178.8	2616.4 ± 946.4	2581.4 ± 1040.6	3131.0 ± 1484.3	2874.2 ± 1600.2	2737.9 ± 1339.7	3163.8 ± 1211.0	3192.2 ± 1481.8	3359.1 ± 1703.9
**Whole day** **(min/16 h day)**									
Sitting Time	629.9 ± 78.7	633.2 ± 91.1	694.8 ± 92.7	589.2 ± 122.6	601.4 ± 110.7	612.5 ± 116.5	623.2 ± 96.6	613.8 ± 147.9	622.1 ± 122.2
Standing Time	227.1 ± 65.3	245.2 ± 90.9	188.4 ± 78.4	266.9 ± 96.4	259.2 ± 79.4	257.1 ± 116.7	224.5 ± 73.0	245.4 ± 140.2	244.5 ± 110.8
Stepping Time	103.0 ± 33.1	81.6 ± 13.0	76.8 ± 22.9	103.9 ± 42.9	99.4 ± 38.1	90.4 ± 24.8	112.4 ± 35.0	100.8 ± 30.4	93.5 ± 28.5
Prolonged Sitting Time (≥30 min)	355.1 ± 103.3	350.5 ± 132.9	420.3 ± 144.2	295.8 ± 123.3	297.2 ± 107.5	331.1 ± 106.9	329.6 ± 107.3	342.3 ± 144.1	379.3 ± 126.6
Steps (number)	8681.6 ± 3349.1	6725.5 ± 1268.2	6320.3 ± 1930.5	8413.3 ± 3715.6	7962.4 ± 325.5	7142.0 ± 2086.3	9946.0 ± 3493.9	8007.7 ± 2353.6	7656.3 ± 2694.6

No-Prompt Comparison arm downloaded the Rise and Recharge smartphone application and received no prompts during the intervention. The Prompt-30 and Prompt-60 Intervention arms downloaded the Stand Up smartphone application and installed prompts at either 30 or 60 min intervals, respectively. Data are presented as the mean ± SD.

**Table 3:** Adjusted mean change and standardised effect size (Cohen’s *d*) between the Prompt-30 and Prompt-60 Intervention arms relative to the No-Prompt Comparison arm.

	Adjusted Mean Change 0–6 Weeks				Adjusted Mean Change 0–12 Weeks			
	Prompt-30 Coefficient (95% CI)	Cohen’s *d* (95% CI)	Prompt-60 Coefficient (95% CI)	Cohen’s *d* (95% CI)	Prompt-30 Coefficient (95% CI)	Cohen’s *d* (95% CI)	Prompt-60 Coefficient (95% CI)	Cohen’s *d* (95% CI)
**Worktime (ActivPAL)** **(min/8 h workday)**								
Sitting Time	−35.5 (−73.8, 3.0)	−0.4 (−1.0, −1.6)	−46.7 (−86.4, −6.6) *	5.6 (5.2, 6.0)	−37.0 (−78.0, 4.2)	−0.5 (−1.3, −2.2)	−69.8 (−111.0, −28.2) **	−20.5 (−20.8, −19.3)
Standing Time	29.4 (−7.2, 66.0)	0.6 (0.3, 0.7)	40.8 (34.8, 8.6) *	−4.7 (−5.0, −4.3)	34.8 (−3.6, 73.8)	0.3 (−0.1, 0.0)	64.8 (25.8, 103.8) **	10.0 (9.4, 10.6)
Stepping Time	6.0 (−6.6, 18.6)	0.4 (0.3, 0.7)	7.1 (−6.0, 20.4)	−2.9 (−3.0, −2.8)	3.0 (−10.2, 16.2)	0.0 (−0.1, 0.1)	7.2 (−6.0, 20.4)	0.8 (0.7, 0.8)
Prolonged Sitting Time (≥30 min)	−7.8 (60.0, 76.2)	0.5 (−0.1, 0.1)	23.4 (−48.0, 95.4)	4.2 (3.6, 4.8)	−27.0 (−99.0, 45.0)	0.0 (−0.7, 1.0)	−25.8 (−98.4, 47.4)	7.2 (6.3, 8.2)
**Whole day** **(min/16 h day)**								
Sitting Time	−7.2 (−63.0, 49.2)	−0.2 (−0.6, −0.9)	−10.2 (−67.8, 47.4)	0.3 (−0.4, 0.9)	−42.0 (−100.8, 16.8)	−0.5 (−1.1, −1.8)	−28.2 (−90.0, 3.8)	3.9 (3.3, 4.5)
Standing Time	−7.2 (−54.6, 40.2)	−0.2 (−0.5, −0.8)	7.2 (−41.4, 55.8)	−0.3 (−0.9, 0.3)	29.4 (−20.4, 79.2)	0.4 (0.0, 0.2)	33.6 (−19.8, 87.6)	−13.5 (−13.9, −13.0)
Stepping Time	14.4 (−48.0, 33.6)	0.8 (0.6, 1.2)	3.0 (−16.8, 22.8)	2.2 (2.0, 2.3)	12.6 (−7.8, 32.4)	0.7 (0.5, 1.1)	−4.8 (−26.4, 16.8)	0.7 (0.5, 0.9)
Prolonged Sitting Time (≥30 min)	−7.2 (−78.6, 64.2)	−0.2 (−0.7, −1.1)	31.2 (−42.0, 44.4)	−0.9 (−1.7, −0.2)	−23.4 (−98.4, 51.6)	−0.5 (−1.1, −1.9)	17.4 (−63.0, 98.4)	−29.0 (−29.9, −28.2)

No-Prompt Comparison arm downloaded the Rise and Recharge smartphone application and received no prompts during the intervention. The Prompt-30 and Prompt-60 Intervention arms downloaded the Stand Up smartphone application and installed prompts at either 30 or 60 min intervals, respectively. Data are presented as the coefficient (95% confidence interval). * Indicates significant at *p* < 0.05. ** Indicates significant at *p* < 0.01. Effect size interpreted as *d* = 0.2 small, *d* = 0.5 medium, and *d* = 0.8 large effect [49].

## Supplementary Materials

The following information is available online at https://www.mdpi.com/1660-4601/17/24/9300/s1: Figure S1: Download and user instructions for the Stand Up and Rise and Recharge smartphone applications; Table S1: Completion rates for outcome measures per time-point; Table S2: Descriptive PA data from ActiGraph GT9X presented by group and time-point; Table S3: Participants mean anthropometric data collected at baseline, 6 and 12-weeks; Table S4: Adjusted mean change and standardised effect size (Cohen’s d) between the Prompt-30 and Prompt-60 Intervention arms, relative to the No-Prompt Comparison arm for anthropometric outcomes; Table S5: Participants mean cardiometabolic data collected at baseline and 12 weeks; Table S6: Adjusted mean change and standardised effect size (Cohen’s *d*) between the Prompt-30 and Prompt-60 Intervention arms, relative to the No-Prompt Comparison arm for cardiometabolic outcomes.
