# Supplementary material for: Rise and Recharge: Effects on Activity Outcomes of an e-Health Smartphone Intervention to Reduce Office Workers’ Sitting Time
Source: Int J Environ Res Public Health. 2020 Dec 12;17(24):9300. doi: 10.3390/ijerph17249300 (PMC7764765; doi:10.3390/ijerph17249300)
Supplement: Supplementary file 1 [file ijerph-17-09300-s001.pdf]

International Journal of
Environmental Research and Public Health

MDPI

Swansea University
Prifysgol Abertawe

Rise and Recharge

Download and user instructions for the Stand Up app

1. Locate the app store on your iPhone

The Stand Up app is only available for download on Apple devices

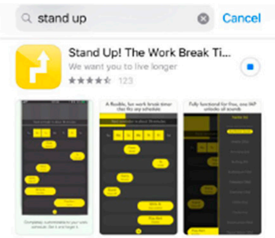

2. Search for the 'Stand Up' app

- Select 'Get'
- This is a free app and you will not be charged to download, although you may be asked to enter your apple password.
- Once you have downloaded the app it will appear on your home screen.

3. Open the app and you will be prompted to receive 'push notifications'.

- In order for you to receive notifications throughout the project you should select 'Allow' as highlighted here in the red circle.
- You can change this in your phone settings at any time.

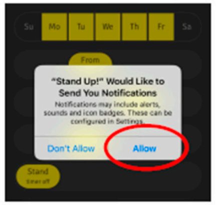

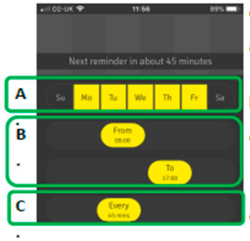

4. Selecting settings

a. Select your working days only e.g. Monday-Friday, by tapping the relevant days.

b. Select your working hours e.g. 'from 9.00 am' 'to 5.00pm'

c. Select every 30 or every 60 minutes depending on which group you have been allocated to for the intervention. If you are unsure please contact a.s.morris@swansea.ac.uk

d. These selections can be modified if your working days or hours vary throughout the week.

5. Selecting settings

a. Throughout the intervention you will be asked to break up your sitting time every 30 or 60 minutes. It is up to you whether you chose to stand up or even take a short walk, for example to speak with a colleague or refill your water bottle.

b. You will not be asked to set a minimum duration for standing so please leave this timer off.

c. You do not need to select a specific location and therefore do not need to turn on your location settings in your iPhone.

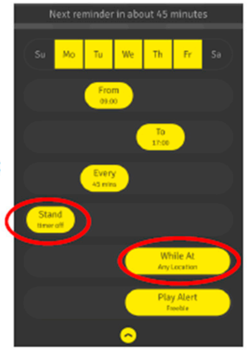

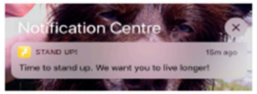

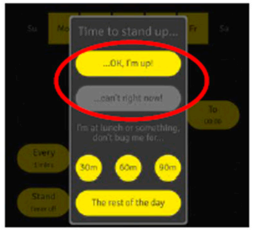

6. Notifications

a. You will receive a notification every 30 or 60 minutes which will pop up on your screen even if your phone is inactive. If your phone is not on silent this will also be accompanied by an alert sound.

b. IMPORTANT: To log your break you will need to open the screen and select one of the two options circled.

c. You may also select whether you would like to postpone the prompt for 30, 60 or 90 minutes.

d. IMPORTANT: If you ignore over 40 notifications during the intervention the app will stop sending you push notifications. Please do not regularly ignore the prompts even I you have been unable to break up your sitting time.

6. Seven day history

a. Select the up arrow (circled) to explore your 7 day history.

b. Each week you will be asked to go onto the 7 day history page and screen shot the summary of results.

c. Please email this screenshot to a.s.morris@swansea.ac.uk each week or send to Abby on TEL XXX

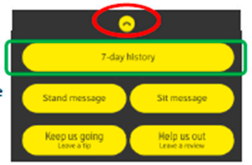

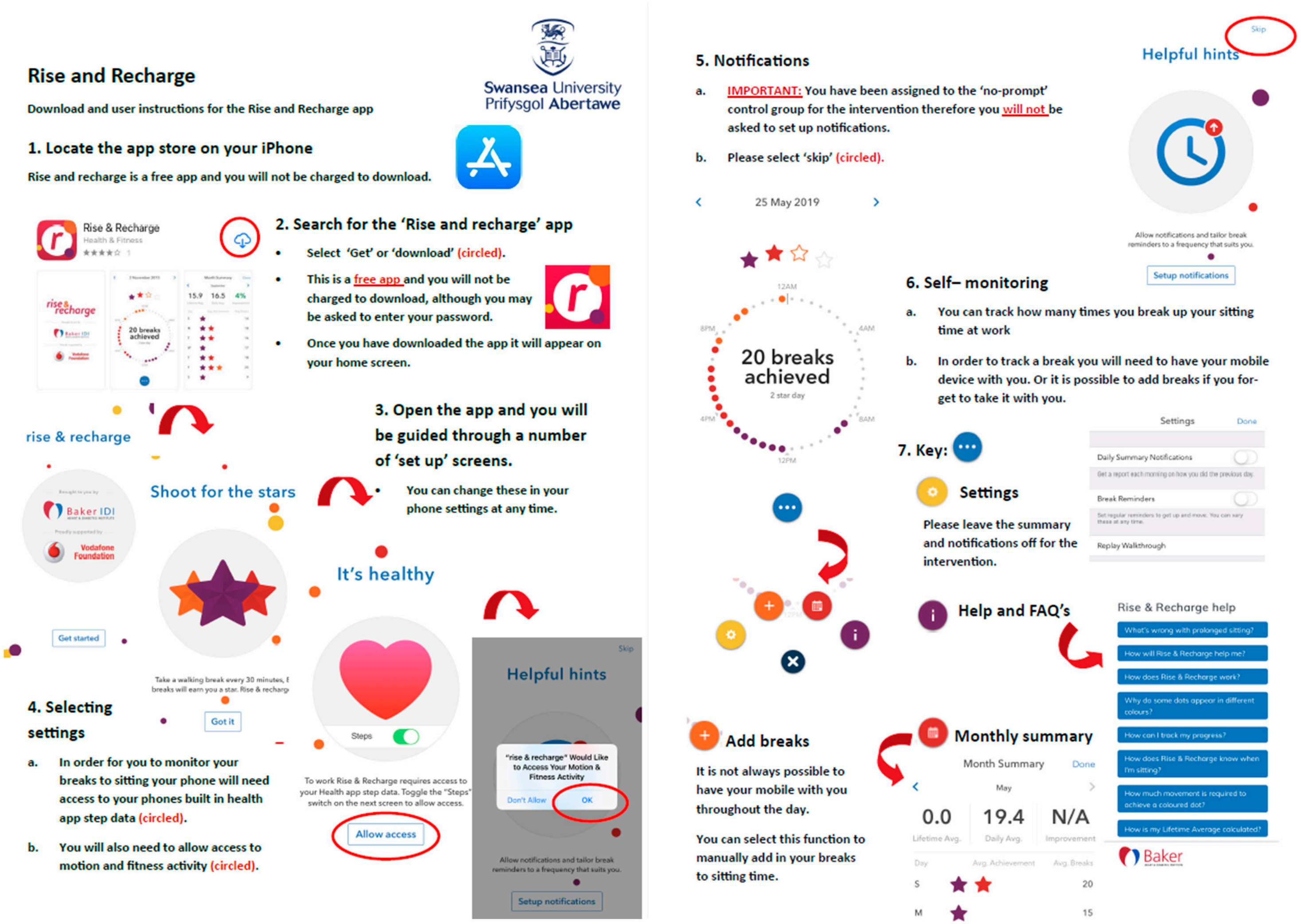

Supplementary Material Figure S1. Download and user instructions for the Stand Up and Rise and Recharge smartphone applications.

Supplementary Material Table S1. Completion rates for outcome measures per time-point.

	**Baseline (*n* = 56)**	**6 weeks (*n* = 51)**	**12 weeks (*n* = 44)**
Behavioural			
ActivPAL (work time)	49 (88)	32 (63)	30 (68)
ActivPAL (whole day)	54 (96)	39 (76)	31 (70)
ActiGraph (whole day)	52 (93)	25 (49)	36 (81)
Anthropometric			
Stature	56 (100)	51 (100)	44 (100)
Body mass	56 (100)	51 (100)	44 (100)
Waist circumference	56 (100)	51 (100)	44 (100)
Hip circumference	56 (100)	51 (100)	44 (100)
Cardiometabolic			
Blood glucose (HBa1c; mmol/L^{-1})	20 (35)	-	17 (39)
Blood glucose (non-fasted; mmol/L^{-1})	49 (88)	-	44 (100)
Total cholesterol (mmol/L^{-1})	56 (100)	-	44 (100)
Systolic and diastolic blood pressure (mmHg)	55 (98)	-	44 (100)
Online questionnaire [b]	52 (93)	29 (57)	21 (48)

No-Prompt Comparison arm downloaded the Rise and Recharge smartphone application and received no prompts during the intervention. Prompt-30 and Prompt-60 Intervention arms downloaded the Stand Up smartphone application and installed prompts at either 30 or 60 minute intervals respectively. [a] Completion rate was the percentage of participants that provided valid data for an outcome measure per time-point out of those engaged in data collection per time point. [b] Online questionnaire includes demographic, EQ5D, Job performance and satisfaction, work limitations, sleep quality index, self-report habit index, workforce sitting questionnaire, employee engagement and musculoskeletal discomfort (data not presented). Data presented as n (%; pooled group data). - Indicates cardiometabolic measures not taken at 6-week data collection.

Supplementary Materials Table S2. Descriptive PA data from ActiGraph GT9X presented by group and time-point.

	No-Prompt Comparison			Prompt-30			Prompt-60		
**Whole Day (mins)**	**Baseline (*n* = 19)**	**6 weeks (*n* = 11)**	**12 weeks (*n* = 13)**	**Baseline (*n* = 20)**	**6 weeks (*n* = 9)**	**12 weeks (*n* = 12)**	**Baseline (*n* = 13)**	**6 weeks (*n* = 5)**	**12 weeks (*n* = 10)**
Light PA	92.3 ± 34.3	81.5 ± 34.3	78.5 ± 31.4	97.6 ± 34.3	96.6 ± 30.8	96.7 ± 31.8	108.3 ± 28.6	96.0 ± 10.4	82.2 ± 28.4
Moderate PA	42.1 ± 13.6	37.8 ± 14.4	38.9 ± 15.3	47.1 ± 17.9	44.3 ± 15.8	47.3 ± 17.3	54.1 ± 19.8	46.2 ± 8.1	42.9 ± 17.2
Vigorous PA	0.4 ± 0.2	0.3 ± 0.2	0.5 ± 0.3	0.8 ± 1.0	0.7 ± 0.6	1.0 ± 1.2	0.9 ± 0.5	0.7 ± 0.4	0.9 ± 0.5
Sleep	368.2 ± 69.6	396.0 ± 63.9	355.2 ± 83.2	353.9 ± 86.9	386.5 ± 49.8	391.6 ± 42.2	359.4 ± 52.3	370.9 ± 39.7	380.8 ± 99.4

No-Prompt Comparison arm downloaded the Rise and Recharge smartphone application and received no prompts during the intervention. Prompt-30 and Prompt-60 Intervention arms downloaded the Stand Up smartphone application and installed prompts at either 30 or 60 minute intervals respectively. Data presented as mean ± SD.

Supplementary Material Table S3. Participants mean anthropometric data collected at baseline, 6 and 12-weeks.

	No-Prompt Comparison			Prompt-30			Prompt-60		
	Baseline (*n* = 21)	6 weeks (*n* = 19)	12 weeks (*n* = 14)	Baseline (*n* = 20)	6 weeks (*n* = 17)	12 weeks (*n* = 15)	Baseline (*n* = 15)	6 weeks (*n* = 15)	12 weeks (*n* = 15)
Body mass (kg)	87.4 ± 18.3	88.9 ± 18.5	91.1 ± 20.0	81.3 ± 18.1	76.6 ± 11.7	76.5 ± 12.4	79.8 ± 24.5	80.1 ± 24.1	80.1 ± 24.3
BMI (kg•m^{-2})	31.1 ± 6.7	29.4 ± 9.6	31.7 ± 7.1	28.4 ± 5.9	26.7 ± 3.5	26.6 ± 3.8	26.9 ± 5.7	31.8 ± 18.5	27.1 ± 5.8
Waist circumference (cm)	95.4 ± 16.7	95.0 ± 15.0	96.4 ± 16.9	88.8 ± 15.4	85.0 ± 9.4	81.3 ± 7.9	83.8 ± 15.7	83.8 ± 15.3	81.7 ± 15.0
Hip circumference (cm)	108.8 ± 16.0	111.8 ± 10.3	110.4 ± 11.3	107.4 ± 10.0	104.2 ± 7.5	102.7 ± 8.5	105.3 ± 14.3	106.3 ± 13.6	104.4 ± 13.6
Waist to hip ratio	0.89 ± 0.16	0.85 ± 0.09	0.87 ± 0.08	0.82 ± 0.09	0.82 ± 0.07	0.79 ± 0.06	0.79 ± 0.09	0.79 ± 0.09	0.78 ± 0.08

No-Prompt Comparison arm downloaded the Rise and Recharge smartphone application and received no prompts during the intervention. Prompt-30 and Prompt-60 Intervention arms downloaded the Stand Up smartphone application and installed prompts at either 30 or 60 minute intervals respectively. Data presented as mean ± SD.

Supplementary Material Table S4. Participants mean cardiometabolic data collected at baseline and 12 weeks.

	No-Prompt Comparison		Prompt-30		Prompt-60	
	Baseline (*n* = 20)	12 weeks (*n* = 14)	Baseline (*n* = 20)	12 weeks (*n* = 15)	Baseline (*n* = 15)	12 weeks (*n* = 15)
Systolic BP (mmHg)	139.3 ± 16.0	135.3 ± 14.1	137.7 ± 17.7	135.0 ± 15.2	132.3 ± 14.5	128.1 ± 11.5
Diastolic BP (mmHg)	91.1 ± 14.8	89.6 ± 12.2	89.8 ± 7.2	85.6 ± 13.7	87.5 ± 10.1	84.9 ± 10.2
Total Cholesterol (mmol•L^{-1})	4.5 ± 1.2	4.4 ± 1.1	4.0 ± 0.8	4.3 ± 0.9	4.2 ± 1.1	4.0 ± 0.8
HDL Cholesterol (mmol•L^{-1})	1.0 ± 0.3	1.2 ± 0.3	1.2 ± 0.5	1.5 ± 0.2	1.2 ± 0.4	1.3 ± 0.4
LDL Cholesterol (mmol•L^{-1})	3.4 ± 1.2	3.2 ± 0.3	2.8 ± 0.8	2.8 ± 0.7	3.0 ± 1.0	2.7 ± 0.8
Cholesterol Ratio (Total Cholesterol: HDL)	4.8 ± 1.9	3.8 ± 1.2	3.7 ± 1.6	3.0 ± 0.5	3.8 ± 2.2	3.4 ± 1.1
Glucose (HBa1C; %)	5.7 ± 0.4	5.7 ± 0.2	5.4 ± 0.3	5.5 ± 0.2	5.6 ± 0.3	5.5 ± 0.4

No-Prompt Comparison arm downloaded the Rise and Recharge smartphone application and received no prompts during the intervention. Prompt-30 and Prompt-60 arms downloaded the Stand Up smartphone application and installed prompts at either 30 or 60 minute intervals respectively. Data presented as mean ± SD.

Supplementary Material Table S5. Adjusted mean change and standardized effect size (Cohen's d) between the Prompt-30 and Prompt-60 Intervention arms, relative to the No-Prompt Comparison arm for anthropometric outcomes.

	Adjusted mean change 0–6 weeks				Adjusted mean change 0–12 weeks			
	**Prompt-30 coefficient (95% CI)**	**Cohen's *d* (95% CI)**	**Prompt-60 coefficient (95% CI)**	**Cohen's *d* (95% CI)**	**Prompt-30 coefficient (95% CI)**	**Cohen's *d* (95% CI)**	**Prompt-60 coefficient (95% CI)**	**Cohen's *d* (95% CI)**
Body mass (kg)	0.2 (−0.9, 1.3)	0.2 (−0.3, 0.7)	−0.2 (−1.3, 1.0)	−0.1 (−0.7, 0.5)	−0.5 (−1.7, 0.7)	−0.3 (−1.0, 0.4)	−0.6 (−1.8, 0.6)	−0.3 (−1.2, 0.6)
Waist to hip ratio	0.0 (−0.0, 0.1)	0.7 (0.62, 0.69)	0.0 (−0.0, 0.1)	0.4 (0.4, 0.5)	0.0 (−0.0, 0.1)	0.5 (0.5, 0.6)	0.0 (−0.0, 0.1)	0.4 (0.4, 0.4)

No-Prompt Comparison arm downloaded the Rise and Recharge smartphone application and received no prompts during the intervention. Prompt-30 and Prompt-60 Intervention arms downloaded the Stand Up smartphone application and installed prompts at either 30 or 60 minute intervals respectively. Data is presented as coefficient (95% Confidence interval). * Indicates significant $p < 0.05$ ** Indicates significant $p < 0.01$. Effect size interpreted as d = 0.2 small, d = 0.5 medium, and d = 0.8 large effect [49].

Supplementary Material Table S6. Adjusted mean change and standardized effect size (Cohen's d) between the Prompt-30 and Prompt-60 Intervention arms, relative to the No-Prompt Comparison arm for cardiometabolic outcomes.

	**Adjusted Mean Change 0–12 weeks**					
	**Prompt-30 coefficient (95% CI)**	***p***	**Cohen's *d* (95% CI)**	**Prompt-60 coefficient (95% CI)**	***p***	**Cohen's *d* (95% CI)**
Systolic BP (mmHg)	3.4 (−3.6, 10.5)	0.34	−0.2 (−9.4, 9.0)	2.2 (−4.9, 9.3)	0.54	−0.3 (−9.4, 8.9)
Diastolic BP (mmHg)	−0.1 (−6.7, 6.6)	0.99	−0.3 (−7.7, 7.2)	1.3 (−5.4, 8.0)	0.70	−0.2 (−7.3, 6.9)
Total Cholesterol (mmol•L^{-1})	0.4 (−0.2, 1.0)	0.15	0.6 (0.2, 0.9)	-0.1 (−0.7, 0.5)	0.82	0.0 −.4, 0.4)
HDL Cholesterol (mmol•L^{-1})	0.3 (−0.3, 0.3)	0.84	−0.0 (−0.2, 0.1)	-0.2 (−0.5, 0.1)	0.26	−0.6 (−0.8, -0.5)
LDL Cholesterol (mmol•L^{-1})	0.4 (−0.2, 1.0)	0.18	0.6 (0.2, 0.9)	0.1 (−0.5, 0.7)	0.70	0.2 (−0.2, 0.6)
Cholesterol Ratio (Total Cholesterol: HDL)	0.4 (−0.8, 1.6)	0.56	0.3 (−0.3, 1.0)	0.7 (−0.6, 1.9)	0.28	0.5 (−0.2, 1.2)
Glucose (HBa1C; %)	0.5 (−0.3, 1.3)	0.26	0.2 (0.1, 0.4)	0.9 (0.0, 1.7)	0.04*	−0.4 (−0.6, -0.2)

No-Prompt Comparison arm downloaded the Rise and Recharge smartphone application and received no prompts during the intervention. Prompt-30 and Prompt-60 arms downloaded the Stand Up smartphone application and installed prompts at either 30 or 60 min intervals respectively. Data is presented as coefficient (95% confidence interval). * Indicates significant $p < 0.05$; ** Indicates significant $p < 0.01$. Effect size interpreted as d = 0.2 small, d = 0.5 medium, and d = 0.8 large effect [48].
